# 

**Published:** 1868-02-01

**Authors:** 


*CONTENTS:*

International Medical Congress .......... 65
Uterine Displacements. By L. A. Babcock, M. D., Freeport, Ill .......... 70
Dislocation of Metatarsal Bones. By H. K. Palmer, M. D., Springfield, Ill .......... 73
Ligation of Innominate Artery. By Moses Gunn, M.D., Chicago .......... 74
FOREIGN CORRESPONDENCE .......... 75
BOOK NOTICES:
On the Signs and Diseases of Pregnancy. By Thomas Hawkes Tanner, M. D., F. L. S., etc .......... 79
The Treatment of the Throat and Lungs by Inhalations. By Emil Siegle, M. D .......... 80
Plastics. By David Prince, M.D .......... 80
Chronic Diseases of the Larynx. By Dr. A. Tobold .......... 80
Condie on Diseases of Children .......... 81
EDITORIALS:
Commencement R. M. C .......... 81
Carson's Synopsis .......... 82
Anatomical Specimens, etc .......... 82
LOOT .......... 82

